# A New Supply of Leeches: The Medical Aspects of Their Use

**Published:** 1915-02-13

**Authors:** 


### A NEW SUPPLY OF LEECHES.

### The Medical Aspects of their Use.

The penetrating influence of the war could hardly be better illustrated than by the experience that it has deprived London of a supply of leeches. During the last three months hospitals and practitioners have appealed in vain to the usual sources of supply. The reply invariably has been that all the stocks were exhausted and that there was little hope of a further supply. With the leech-areas of France and Central Europe crowded with armies, the collection of leeches had become a matter of very small importance to the inhabitants, and the expectation of any renewal of the industry seemed to be dependent on the re-establishment of peace. Hence even so uninviting a bargain as a second-hand leech could not be obtained in London either for love or money.

### The Leech from India.

Fortunately, expert knowledge and enterprise have at last managed to meet the position, and we are once more in enjoyment of an abundant leech-supply. This happy state of matters we owe to Professor Shipley, of Cambridge, who has come to the rescue in truly gallant fashion. After unsuccessful appeals to Canada and the United States he turned to India, and his efforts were seconded by Dr. Annandale, Director of the Indian Museum at Calcutta, and by Colonel Alcock, M.D., of the London School of Tropical Medicine. It was under these distinguished auspices that a few days ago a parcel of leeches reached London and was placed in the appropriate keeping of a firm of well-known pharmacists. Possibly the critics may not be quite happy when they learn that the new-comers cannot claim to be exactly the leech of former years. This well-established friend, it seems, enjoyed Pharmacopœial recognition and rejoiced in the name of *Hirudo medicinalis*. The new arrival from the Far East is of another genus and species, and must be spoken of respectfully as *Limnatis granulosa*.

### Supply from Spain.

Another admission that has to be made is that the *Limnatis* is of smaller size and less cubic capacity than his predecessor. All the same he is heartily welcome. He comes with excellent credentials from his Indian home, and judged by his behaviour in a new latitude he is full of energy and equal to any duty to which he may be summoned. This is indeed a very satisfactory issue, and doctors and patients owe much gratitude to Professor Shipley and his colleagues. It is at the same time right to say that we have knowledge of the recent arrival in London of a supply of leeches from Spain, and these happen to be the true and genuine *Hirudines* of the British Pharmacopœia. Hence there is here a chance for those who prefer the ancient ways and who are sticklers for old friendships and official orthodoxy. Remembering the late famine we are disposed to adopt the motto which is attributed to H.M.S. *Lion* and which reads in the vernacular, "Let 'em all come."

### A Defence of Leeching.

We took occasion in our Notes last week to record that the new Pharmacopœia recognised a leech found in the Australasian Colonies; and then remarked on the decline in favour which the use of the leech has suffered of late years. We pointed out that critics declared their use as difficult to reconcile with modern asepsis. Having, therefore, given the devil his due, it is worth while to see what medical case can be made out for them, and to give an instance in which they may still be held to be of striking value.

It has been somewhat of a surprise to the public, and even to some members of the profession, to learn that there existed a demand for leeches so large as has been revealed by the recent scarcity. Of course the scale on which they are employed is trifling compared with the records of former days. Still, it is evident that they are used in considerable numbers, and it would seem that they are employed much more largely in the hospitals than in private practice. In this fact there appears a suggestion that possibly their use is too largely neglected.

### Empirical Therapy.

Indeed, with so many new-fashioned methods competing for attention there may be danger of undervaluing older practices. Again, it must be admitted that leeching is anything but an æsthetic procedure, and the revolt from excess is apt to condemn it as out of date and even barbarous. There is still another point. In the modern atmosphere there is so insistent a demand and passion for the rational that measures having only an empirical basis are often treated as hopelessly unscientific and altogether behind the times. The use of leeches is often brought under this condemnation, and superior persons turn from it with a disdainful shrug. The truth is that, whatever be the explanation, there are certain inflammatory and congestive conditions in which the use of leeches is often strikingly beneficial. One of the most impressive demonstrations of the truth of this proposition is in the treatment of iritis and other inflammatory affections of the eyeball. No ophthalmic surgeon would deprive himself of this resource, and what is true in relation to the eyeball is surely likely to be true in reference to other organs. Take, again, the embarrassed breathing due to a failing right ventricle, and no one who has made the experiment will doubt how much relief can be obtained by the free use of leeches. In our experience they are a valuable therapeutic resource, and it is therefore with much satisfaction that we learn of a re-establishment of the supply.

